# Opioid Use Following Cesarean Delivery: A Pilot Study on Patterns of Use, Storage, and Disposal

**DOI:** 10.7759/cureus.49474

**Published:** 2023-11-27

**Authors:** Dara Seybold, Kelly Simmons, Lesli A Taylor, Annie R Roslonski, Blake Rozycki, Byron Calhoun

**Affiliations:** 1 Obstetrics and Gynecology, Institute for Academic Medicine, Charleston Area Medical Center, Charleston, USA; 2 Obstetrics and Gynecology, West Virginia University, Charleston Division, Charleston Area Medical Center, Charleston, USA; 3 Charleston Area Medical Center, Institute for Academic Medicine, Charleston, USA; 4 Maternal-Fetal Medicine, West Virginia University, Charleston Division, Charleston, USA

**Keywords:** obstetrics care, opioid use, post-operative pain management, substance use obstetrics, cesarean birth

## Abstract

Objective

The aim of this study was to describe various aspects related to opioid use and storage in the setting of at-home pain management after cesarean deliveries among an Appalachian population.

Methods

Women who underwent cesarean delivery (January-June 2019) at an Appalachian institution were prospectively enrolled and administered a telephone survey seven (± 3) days post-discharge.

Results

Of the 87 women enrolled, 40 (46%) completed the survey; 92.5% were prescribed an opioid medication, most commonly oxycodone/acetaminophen 5/325 mg. A Kruskal-Wallis H test revealed a significant association between the severity of pain that interfered with normal daily activities and the number of pills consumed [χ2(2)=6.75, p=0.034]. More than 70% of the participants (28/40) had not safely stored or disposed of their unused opioid medications.

Conclusion

Our findings highlight the need for interventions to educate patients on how to appropriately use, store, and dispose of unused opioids.

## Introduction

The cesarean rate in the US has been reported to be around 33%, and several women are undergoing major surgery to have a child. In fact, cesarean surgery is the most common inpatient surgical procedure in the US [1]. There is no standard regimen for pain management after cesarean. While opiates are commonly prescribed, they are often overprescribed due to the variation in patient needs. In a survey-based qualitative study, 84% of women reported overprescription of opioids after cesareans [2]. Another retrospective cohort study found that 87% of postpartum women were discharged with an opioid prescription following cesarean delivery and their dose did not vary based on pain they reported while in the hospital [3]. One of the factors associated with increased opioid use is the higher number of opioid tablets prescribed [4]. When there is a mismatch between the amount of opioid prescription and the pain requirements, and if, subsequently, there are leftover pills, misuse or drug diversion can occur [5,6]. The rates of leftover opioids can be very high. A prospective observational cohort study reported that 75% of patients had unused opioids at their homes, and 63% were storing them in an unlocked location [7].

The opioid epidemic is an ongoing public health issue in the US, and the population in the Appalachian region has been significantly affected by this crisis. Currently, West Virginia has the highest rate of drug overdose deaths associated with opiate use when compared with other states [8]. Up to 70% of people who use opioid medications that were not prescribed to them had received them from family or friends [6,9,10]. Obstetricians and gynecologists can help combat this epidemic by improving practices related to pain management. Prescribing appropriate pain medication should be done with great care, ensuring that opioids are indicated for the patient's condition and that the risks and benefits of opioid use are discussed in great detail with the patient [11]. In the last few years, attempts have been made to address opioid addiction and overuse by introducing the practice of maximum tablet count for opioid prescriptions [12], educating providers on opioid prescribing habits, and implementing an individualized patient pain management plan [5,11].

The American College of Obstetrics and Gynecology‘s most recent stepwise multimodal approach involves beginning treatment with acetaminophen and/or non-steroidal anti-inflammatory drugs (NSAIDs) and then escalating to opioids for breakthrough pain [10,13] and educating women about their own and their breastfed infant’s central nervous system risks regardless of the type of opioid prescribed [14]. The stepwise multimodal pain management approach has been associated with reduced opioid consumption [15] without an increase in reported pain or length of hospital stay [16]. Furthermore, reducing the amount of prescribed opioids may prevent the diversion of medications to others as leftover pills are often improperly stored with easy access to others. In 2016, an article in the Journal of American Medical Association highlighted the need for additional research to devise effective and safe practices regarding opioid medication sharing, storage, and disposal [6]. The purpose of this study was to discuss the current practices related to physicians' prescription of opioids as well as patient use, storage, and disposal of leftover opioids pertaining to postoperative at-home pain management after cesarean sections among an at-risk Appalachian population.

## Materials and methods

Study design

This was a prospective cohort study involving women managing pain after a cesarean delivery. Women who delivered via cesarean sections between January through June 2019 and provided informed consent to participate in this research study were included. Study staff documented the best way for study participants to be contacted after being discharged from the hospital so that the follow-up questionnaire (Appendix A) could be administered. Study variables were collected, including pain management instructions with the type and amount of pain medicine prescribed. Study participants were telephoned seven (± 3) days after their date of discharge to complete a telephone survey (Appendix A) regarding the amount and duration of opioid use, information on opioid storage, and whether other means were used to manage pain.

Selection criteria

Consecutive patients who met the following inclusion/exclusion criteria were asked to participate in the study: inclusion criteria: age of 18-50 years, delivered liveborn via cesarean at the study hospital without general anesthesia; exclusion criteria: a history of chronic pain or chronic opioid use including methadone and buprenorphine, positive drug screening at the time of labor and delivery, postoperative hospitalization <5 days, contraindication to acetaminophen or NSAIDs, undergoing additional surgeries such as tubal ligation and hysterectomy.

Statistical analysis

Data were analyzed with IBM SPSS Statistics Version 19.0 (IBM Corp., Armonk NY). Descriptive statistics and univariate analysis were used as appropriate to assess continuous and categorical variables. Continuous variables were presented as means and standard deviations (SD) and were compared using independent samples t-test while categorical variables were reported as percentages and compared using Chi-square or Fisher’s exact test. A Kruskal-Wallis H test was performed to test for a relationship between patient-perceived pain interfering with normal daily activities and the volume of opiate pain pills consumed.

## Results

Of the 87 women enrolled, 40 (46%) responded to the telephone survey and answered all questions, including seven (8.%) patients who did not fill out their opiate prescriptions. The mean maternal age was 27.6 ±5.7 years, and the majority (82; 94.3%) were Caucasian; 38 (43.7%) were covered by Medicaid. The mean BMI of the cohort was in the obese range (37.4 ±8.8). There were no statistically significant differences in maternal characteristics between the "response" and "no response" groups in terms of the aforementioned variables and other characteristics (Table 1).

**Table 1:** Maternal characteristics by survey completion BMI: body mass index; SD: standard deviation

Maternal characteristics	Total, n=87	Response, n=40	No response, n=47	P-value
Age, years, mean ±SD	27.6 ±5.7	27.2 ±6.0	27.9 ±5.5	0.600
Ethnicity/race, n (%)				0.389
White	82 (94.3)	38 (95.0)	725 (35.2)	
African American	4 (4.6)	1 (2.5)	3 (6.4)	
More than one	1 (1.1)	1 (2.5)	0 (0.0)	
Medicaid, n (%)	38 (43.7)	16 (40.0)	22 (46.8)	0.665
Gravidity, mean ±SD	2.1 ±1.4	2.0 ±1.2	2.3 ±1.6	0.261
Nulliparous, n (%)	40 (46.0)	21 (52.5)	19 (40.4)	0.287
BMI, mean ±SD	37.4 ±8.8	36.2 ±6.6	38.3 ±10.1	0.294
Hypertension, n (%)	21 (24.1)	9 (22.5)	12 (25.5)	0.805
Diabetes, n (%)	9 (10.3)	2 (5.0)	7 (14.9)	0.142
Tobacco use, n (%)	6 (6.9)	3 (7.5)	3 (6.4)	1.000
Substance use, n (%)	6 (6.9)	2 (5.0)	4 (8.5)	0.683

These cesarean deliveries were characterized by a 77.5% (31/40) rate of analgesic use; 10% (4/40) had epidural anesthesia; 90% (35/40) had spinal anesthesia; 50% (20/40) were administered fentanyl; and 55% (22/40) were administered morphine (Table 2).

**Table 2:** Cesarean delivery characteristics

Characteristics	N (%)
Analgesic	
Yes	31 (77.5)
No	9 (22.5)
Type	
Scheduled	27 (67.5)
Unscheduled	13 (32.5)
Anesthesia	
Spinal	36 (90.0)
Epidural	4 (10.0)
Fentanyl	
Yes	20 (50.0)
No	20 (50.0)
Morphine	
Yes	22 (55.0)
No	18 (45.0)

Most patients were prescribed Percocet (oxycodone/acetaminophen) 5/325mg (35/40; 87.5%), followed by no opioids (3/40; 7.5%), and oxycodone (2/40; 5.0%). In terms of dosage, 20 tablets were the most common quantity prescribed (30/40; 75%). Almost all patients used either prescription strength (16/40; 40%) or over-the-counter (OTC) ibuprofen (22/40; 55%), with one using Tylenol and only one using no other pain medicine. On a pain scale of 0-5, most of the women ranked their pain between 0-2 (25; 62.5%) with a mean of 2.15 ±1.9. When asked about pain interfering with normal daily activity, patient responses ranged from 1-4 on a scale of 0-10, with a mean score of 2.7 ±0.82. Table 3 provides more details on survey responses.

**Table 3:** Telephone survey response on pain management post-cesarean section delivery after hospital discharge (n=40) OTC: over-the-counter

Characteristics of post-cesarean section pain management at home	N (%)
Filled prescription	
Yes	33 (82.5)
No	7 (17.5)
Type of opioid	
Percocet	35 (87.5)
Oxycodone	2 (5.0)
None	3 (7.5)
Number of pills prescribed	
28	1 (2.5)
24	4 (10.0)
20	30 (75.0)
15	1 (2.5)
10	1 (2.5)
0	3 (7.5)
Use of other pain medicine	
Prescription ibuprofen	16 (40.0)
OTC ibuprofen	22 (55.0)
Tylenol	1 (2.5)
None	1 (2.5)
Pain scale score (0-5)	
8	1 (2.5)
5	4 (10.0)
4	4 (10.0)
3	6 (15.0)
2	8 (20.0)
1	8 (20.0)
0	9 (22.5)
Pain interfering with daily activity (score on a scale of 1-10)	
10	0 (0.0)
9	0 (0.0)
8	0 (0.0)
7	0 (0.0)
6	0 (0.0)
5	0 (0.0)
4	8 (20.0)
3	13 (32.5)
2	18 (45.0)
1	1 (2.5)
0	0 (0.0)
Plans for unused opioid pills	
Store unsecured	15 (37.5)
Discard (trash, flush, return to pharmacy)	10 (25.0)
Not sure	3 (7.5)
None left/not applicable	12 (30.0)

A Kruskal-Wallis H test revealed that there was a significant association between the severity of pain that interfered with patients’ normal daily activities and the number of opiate pain pills they consumed: χ2(2)=6.75, p=0.034 (Table 4).

**Table 4:** Relationship between severity of pain interfering with normal activities and opioid use

	Interference, mean rank number of tablets		
	Minimal activity interference	Moderate activity interference	Significant activity interference
Number of opioid tablets used	15.76	23.15	27.44

There was no difference among patients in terms of the extent to which their pain interfered with their activities, such as "a little bit", "moderately", or "quite a bit". Most importantly, we discovered that patients were not safely storing or disposing of opioid medication. Of the 40 patients, 12 (30%) had no pills left at the time of the interview, leaving a total of 344 unused pills among 28 patients. While 3/28 (10.7%) patients were unsure as to what they were going to do with the leftover pills, only 10/28 (35.7%) patients reported plans to dispose of their medications appropriately. Appropriate disposals reported by patients involved using medication disposal bags, flushing the pills down, or returning them to the pharmacy. The remaining 15/28 (53.6%) patients were storing their medications in unlocked locations such as purses, kitchen counters, and medicine cabinets, accounting for a total of 192 pills left unsecured in locations that may be accessed by others. This amounted to an average of 12.8 leftover pills (192/15) per patient.

## Discussion

It is very difficult to predict the severity of pain a patient might experience postoperatively and the amount of pain pills the patient will require [3]. There have been many attempts to address this issue, including shared decision-making and individualized opioid prescriptions based on in-hospital opioid use although this is a labor-intensive process [17,18]. However, the natural course of postpartum pain has not been studied or described in a detailed manner and each person experiences pain differently. While there is no standardized pain protocol after cesarean delivery, recent studies advocate for stepwise multimodal approaches and Enhanced Recovery After Surgery (ERAS®) protocols [19,20]. By employing a combination of agents with various mechanisms of action, pain can be optimally treated with reduced opioid use, misuse, and diversion while simultaneously increasing patient satisfaction [5,20]. However, despite these efforts and improvements, the problem still persists and patients are routinely prescribed more opioids than required during the postoperative period, which they are not safely storing or disposing of. Improper disposal and storage increase the risk of family or friends accidentally consuming harmful opioids or intentionally misusing them. This study analyzes the pattern of opioid use, storage, and disposal in the setting of postoperative pain management at home after a cesarean delivery in an Appalachian population.

In our study, patients who experienced pain interfering with their normal daily activities reported consuming more pain pills. This, coupled with insignificant results for all other factors, has enabled us to understand why prescribing an appropriate amount of pain medication is difficult to predict and should be done with great care. Our study has certain limitations, primarily its response rate of under 50%; moreover, the external validity of our findings may be limited due to the fact that our participants were recruited from a single tertiary medical center. Likewise, since we employed a self-reporting survey design for the study, our findings may have been influenced by nonresponse bias and recall bias. However, despite these limitations, this study allowed our institution to obtain baseline data on how our patient population handles prescription drugs. In a geographical area significantly affected by the opioid epidemic, it is critical that we approach the problem carefully by looking at all facets, including addressing the issues of the storage and disposal of unused medications.

## Conclusions

Takeaways from our study include the recommendation for providing opioid disposal bags to patients post-cesarean delivery before discharge, which would enable them to dispose of the medications in an appropriate way and avoid storing pills to be used later in an off-label manner or diverted. In addition, distributing an educational pamphlet focusing on this specific population can provide a concise and understandable summary of proper medication use following this type of surgery, including how to use prescription or OTC ibuprofen as first-line pain management, so that prescribed opioids could be reserved for breakthrough pain. This educational tool could also explain the benefits and risks of opioid use to manage pain and alternative methods to manage pain such as heat therapies (hot showers or heating pad/bottle), cold therapy with ice, and massages, as well as the significance of ample social support in the postpartum period.

These interventions would enable patients to better understand how to control their postoperative pain and choose the correct methods of disposing of pain medications. Future research could evaluate how often patients utilize these prescription disposal bags and potentially expand these findings to other types of surgeries and procedures where addictive medication is dispensed to patients.

## Appendices

Appendix A: Telephone Questionnaire

Introduction: Thank you for volunteering to take part in this study on pain management after cesareans. 

Questions

1. Did you fill your prescription? Y/N

2. How many pills did you take? _____

3. On what day did you stop taking them (Day 1 is the first day at home) _____

(If the patient is still taking, will plan a follow-up call 1 week later)

4. How would you rate your pain today on a scale of 0 (no pain) to 10 (the worst pain possible)?

(0 1 2 3 4 5 6 7 8 9 10). Circle one.

5. What did you do with any leftover pills? _____________

6. Did you use other pain medication? Y/N

If yes, which type of pain medication did you use? __________

7. Did you do anything else to manage your pain? Y/N

a. If yes, what did you do? ___________________

8. Since you left the hospital (about 1 week ago), how often did pain interfere with your normal daily activities? 0 being never at all and 10 being constant everyday interference. _______________

9. Do you have someone with you that helps take care of the baby? Y/N

If yes, how many hours a week do they spend with you? _______ hours

10. Do you have any other children currently residing with you? Y/N

a. If yes, how many children? __________ 

Thank you for taking the time to complete this questionnaire.
